# Polyethylene Terephthalate May Yield Endocrine Disruptors

**DOI:** 10.1289/ehp.0901253

**Published:** 2009-11-25

**Authors:** Leonard Sax

**Affiliations:** Montgomery Center for Research in Child and Adolescent Development, Exton, Pennsylvania, USA

**Keywords:** antimony, bottled water, endocrine disruptors, leaching, phthalates, polyethylene terephthalate

## Abstract

**Background:**

Recent reports suggest that endocrine disruptors may leach into the contents of bottles made from polyethylene terephthalate (PET). PET is the main ingredient in most clear plastic containers used for beverages and condiments worldwide and has previously been generally assumed not to be a source of endocrine disruptors.

**Objective:**

I begin by considering evidence that bottles made from PET may leach various phthalates that have been putatively identified as endocrine disruptors. I also consider evidence that leaching of antimony from PET containers may lead to endocrine-disrupting effects.

**Discussion:**

The contents of the PET bottle, and the temperature at which it is stored, both appear to influence the rate and magnitude of leaching. Endocrine disruptors other than phthalates, specifically antimony, may also contribute to the endocrine-disrupting effect of water from PET containers.

**Conclusions:**

More research is needed in order to clarify the mechanisms whereby beverages and condiments in PET containers may be contaminated by endocrine-disrupting chemicals.

Polyethylene terephthalate (PET) is the material most commonly used to make the clear plastic bottles in which bottled water is sold. PET bottles are also in widespread use as containers for soda beverages, sports drinks, and condiments such as vinegar and salad dressing. PET bottles are also commonly used for the packaging of cosmetic products, such as shampoo, particularly when such products are sold in clear plastic bottles.

The potential of plastic packaging to introduce endocrine disruptors into foods and beverages has gone largely unrecognized until quite recently (Muncke 2009). The plastics industry generally asserts that PET bottles are not a source of endocrine disruptors (e.g., American Chemistry Council 2009). In this commentary, I present evidence that PET bottles may leach endocrine disruptors, and I consider the conditions under which this leaching may occur.

## Synthesis of PET

The synthesis of PET begins with the esterification of either terephthalic acid or dimethyl terephthalate with ethylene glycol, yielding bis(hydroxyethyl)terephthalate (BHET). The BHET is then polymerized up to about 30 repeat units (Awaja and Pavel 2005). Next, to achieve a degree of polymerization (DP) of about 100 repeat units, polycondensation is performed at temperatures > 270°C and pressures > 50 Pa (Ravindranath and Mashelkar 1986). To produce bottle-grade PET, the DP must be > 150 repeat units, which is typically accomplished via solid-state polymerization, a process that requires temperatures > 200°C, pressures > 100 Pa, and incubation times of at least 15 hr (Al-Ghatta et al. 1997).

It is becoming increasingly common for manufacturers to market copolymers for purposes previously filled by homopolymer PET. Copolymer blends, such as polybutylene terephthalate/PET, have certain advantages over homopolymer PET with regard to mechanical properties and resistance to degradation (Grossetête et al. 2000; Guerrica-Echevarría and Eguiazábal 2009). In the United States, a clear plastic bottle may be made with copolymers and still be legally marketed as PET, according to applicable federal regulations [e.g., 21 CFR §177.1630 (U.S. Food and Drug Administration 2009).

## Phthalates

The term “phthalates” refers to the diesters of 1,2-benzenedicarboxylic acid, better known as phthalic acid. A growing literature links many of the phthalates with a variety of adverse outcomes, including increased adiposity and insulin resistance (Grün and Blumberg 2009), decreased anogenital distance in male infants (Swan et al. 2005), decreased levels of sex hormones (Pan et al. 2006), and other consequences for the human reproductive system, both for females and males [reviewed by Hauser and Calafat (2005)]. Infants and children may be especially vulnerable to the toxic effects of phthalates (Sathyanarayana 2008). Indeed, legislatures and government agencies in Australia, Canada, the European Union, and the United States have already acted to restrict or prohibit the use of phthalates in consumer products (Canadian Department of Health 2009; U.S. Consumer Product Safety Commission 2008).

The plastics industry has been keen to emphasize the distinction between PET and phthalates. In a letter to *Environmental Health Perspectives*, a spokesperson for the American Plastics Council wrote:

Plastic beverage bottles sold in the United States are made from a type of plastic known as polyethylene terephthalate (PET). Although polyethylene terephthalate (the plastic) and phthalate (the additive) may have similar names, the substances are chemically dissimilar. PET is not considered an orthophthalate, nor does PET require the use of phthalates or other softening additives. (Enneking 2006)

Indeed, phthalates are not used as substrates or precursors in the manufacture of PET. However, as discussed below, several reports suggest that phthalates may leach from PET bottles into the contents of the bottle.

In this commentary, I first review evidence from various bioassays that PET may yield endocrine disruptors. I then consider evidence that phthalates leach from PET bottles, followed by evidence that antimony leaches from PET bottles.

## Bioassays

Wagner and Oehlmann (2009) employed two bioassays to investigate the estrogenicity of water within PET bottles. First, they used a yeast estrogen screen, employing a strain transfected with the human estrogen receptor α. They evaluated 20 brands of mineral water, nine of which are available both in glass and in PET bottles. Three of nine brands sampled in glass demonstrated significant estrogenic activity in this bioassay, compared with seven of nine brands of water from PET bottles. However, one cannot say with certainty that the estrogenic substance or substances necessarily leached from the bottles; the contaminant may have been introduced into the water before bottling.

The second bioassay by Wagner and Oehlmann (2009) addressed this concern. These investigators emptied the bottles of their contents, then filled the empty PET bottles and glass bottles with a defined culture medium (pH 8.0 ± 0.5) and incubated New Zealand mudsnails, *Potamopyrgus antipodarum*, for 56 days. Production of embryos was significantly enhanced among snails incubated in the PET bottles compared with snails incubated in glass bottles, across all brands (*p* < 0.001). For example, production of embryos incubated in PET bottles of brand D was roughly double the production of embryos incubated in glass bottles of brand D; curiously, on the yeast estrogen screen, this same brand showed no difference in estrogenic activity between PET-bottled water and glass-bottled water. This finding suggests that the *in vivo* snail bioassay might be more sensitive than the *in vitro* yeast estrogen screen.

Regarding the snail bioassay, Wagner and Oehlmann (2009) concluded that “it is obvious that the observed effects can only be attributed to xenoestrogen leaching from these plastic bottles. Moreover, the compounds released by the PET material were potent [enough] to trigger estrogenic effects *in vivo* similar to those of E_2_ [17α ethinyl estradiol] at a concentration of 25 ng/L.” The maximum estrogen activity that they detected in any brand of water was equivalent to 75 ng/L of ethinyl estradiol.

Pinto and Reali (2009) also employed a yeast bioassay to investigate estrogenic activity in samples of water obtained from PET bottles. Like Wagner and Oehlmann (2009), they found large variations among brands of water obtained from PET bottles: In ethinyl estradiol equivalents, their results ranged from a low of 0.9 ng/L to a high of 23.1 ng/L. Pinto and Reali explained this variation by noting that “not all PET materials are of the same chemical quality. Quality may vary depending on the raw material as well as the technology used in bottle production.” Pinto and Reali, using waters bottled in Italy, obtained results that were substantially lower in estrogenic activity than those obtained by Wagner and Oehlmann, who purchased their bottled waters in Germany. It is possible that the German PET had a greater propensity to leach endocrine disruptors than the Italian PET; it is also possible that the yeast bioassay employed by Pinto and Reali may be less sensitive than the yeast bioassay employed by Wagner and Oehlmann.

In a third report, investigators at the University of Missouri tested the effect of an unspecified brand of PET-bottled water on the proliferation of breast cancer cells. They found that the PET-bottled water triggered a 78% increase in the growth of the breast cancer cells compared with the control water: 1,200 breast cancer cells multiplied to 32,000 in 4 days when incubated in PET-bottled water, versus 18,000 for the control sample (Naidenko et al. 2008). Naidenko et al.’s report must be interpreted with caution because it is available only as a posting on the Web and has not yet been published in any peer-reviewed journal. Furthermore, the results from the University of Missouri bioassay, like those from Pinto and Reali (2009), do not prove unequivocally that the presumptive endocrine disruptors leached from the PET bottle wall; they might conceivably have been present in the water before bottling.

## Presence of Phthalates in PET-Bottled Water, Soda, and Food Simulants

Montuori et al. (2008) tested 71 commercial brands of bottled water, all of which were available both in glass bottles and in PET bottles. Across all brands, they found that the concentration of all phthalates combined was “more than 12 times higher in PET than in glass bottled water.” In most cases, the concentration of phthalates in water from glass bottles was below the limits of detection. The most abundant phthalates that they found in PET-bottled water were dibutyl phthalate, diisobutyl phthalate, and diethyl phthalate (DEP). The 50th percentile for the sum of all phthalates in all PET bottles tested by Montuori et al. was 1.32 μg/L. Montuori et al. (2008) asserted that the phthalates they detected in the PET-bottled water must have leached from the PET bottle wall; however, their data do not compel this conclusion, because they did not measure the concentration of phthalates as a function of time. It is conceivable that the water in the PET bottles was contaminated with phthalates before bottling.

Casajuana and Lacorte (2003) investigated the effect of prolonged incubation on the concentration of various phthalates in water from PET bottles compared with water from glass bottles. In all their samples, both from glass bottles and from PET bottles, the concentration of phthalates was initially very low, at or below the limits of detection in almost every case, when first sampled. Prolonged incubation had little effect on the concentration of phthalates in glass-bottled water; phthalates in water from glass bottles were still generally undetectable after 10 weeks of storage. However, in their samples of water from PET bottles, three out of five brands showed measurable levels of di-(2-ethylhexyl) phthalate (DEHP) after 10 weeks of incubation, with an average DEHP concentration of 0.134 μg/L, and all five brands showed measurable levels of DEP after 10 weeks of incubation, with an average DEP concentration of 0.214 μg/L.

Schmid et al. (2008) sought to determine whether solar water disinfection (SODIS) would promote leaching of phthalates into water in PET bottles. SODIS is a technique used in developing countries to disinfect water by incubating water in PET bottles in direct sunlight. After 17 hr of incubation in direct sunlight, maximum concentrations of di(2-ethylhexyl)adipate and DEHP were 0.046 and 0.71 μg/L, respectively.

Biscardi et al. (2003) went to a bottling plant in order to obtain mineral water before bottling. They then filled PET bottles and glass bottles with mineral water, both carbonated and noncarbonated. All bottles were stored at room temperature. Each subsequent month, for 12 months, samples of water were lyophilized, the powders then shaken with acetone, and the acetone extracts analyzed using gas chromatography/mass spectrometry (GC/MS). Throughout the first 8 months, no phthalates were detected in any sample. Beginning at month 9 for PET-bottled noncarbonated water, and month 10 for PET-bottled carbonated water, the acetone extracts increased from 0.4 to > 3.0 mg/L. GC/MS analysis of the extracts identified the presence of DEHP.

Farhoodi et al. (2008) studied the interaction of incubation time with storage temperature on the leaching of DEHP from PET bottles. Using a solution of 3% acetic acid as a food simulant, they incubated the solution in PET bottles for up to 120 days, either at 25°C or at 45°C. On day 0, at the beginning of the trial, the amount of DEHP in PET bottles was below the limits of detection. On day 25, the amount of DEHP in the solution incubated at 25°C was 1.2 mg/L, whereas the amount of DEHP in the solution incubated at 45°C was 2.1 mg/L. By day 66, the amount of DEHP in the solution incubated at 25°C had peaked at 1.4 mg/L, whereas the amount of DEHP in the solution incubated at 45°C had plateaued at 2.5 mg/L.

Bošnir et al. (2007) sought to determine how the contents of the PET bottle influenced the concentration of phthalates in the contents. They compared the concentrations of various phthalates in PET-bottled mineral water with PET-bottled soft drinks preserved with phosphoric acid or with sodium benzoate and/or potassium sorbate. They reported large variations in the concentrations of phthalates both across beverages and across manufacturers. For example, they were not able to detect dimethyl phthalate (DMP) in any brand of mineral water after a 30-day incubation, whereas DMP was the most abundant phthalate detected in the soft drinks they tested. Among soft drinks preserved with both sodium benzoate and potassium sorbate, the concentration of DMP in samples incubated for 30 days ranged from 18 to 2,666 μg/L, with a mean of 501 μg/L; by contrast, the concentration of DMP in mineral water was consistently below the limits of detection. They conjecture that the lower pH of the soft drinks might account for this difference. However, the concentrations of DEHP (unlike DMP) did not differ between soda beverages and mineral water: They found average levels of DEHP < 100 μg/L in all their specimens, with no significant difference between soda beverages and mineral water.

Bošnir et al. (2007) asserted that leaching of phthalates from the PET bottle must be the source of the phthalates they measured; they assumed that the concentration of phthalates was below the limits of detection when the mineral water or soft drinks were originally bottled. Their basis for this assumption is the fact that “raw materials for soft drinks and final products (both soft drinks and mineral water) are under obligatory and continuous public health validity control [in Croatia] which excludes possible contamination with phthalates.” However, they provide no measurements to support their assumption that the soft drinks and mineral water they tested were phthalate-free when bottled. Once again, as with Montuori et al. (2008), one cannot exclude the possibility that the water or soft drinks may have been contaminated with phthalates before bottling.

## Origin of Phthalates in PET-Bottled Water and Beverages

Farhoodi et al. (2008) were not able to detect DEHP in their samples when first tested, yet after 66 days of incubation at 45°C, the concentration of DEHP in their sample reached 2.5 mg/L (i.e., 2,500 μg/L). Likewise, Biscardi et al. (2003) reported similar concentrations of DEHP after a 9-month incubation of water in PET bottles at room temperature. Where did the DEHP come from? That is, how did DEHP get into the PET bottle wall in quantities sufficient for such an amount to leach into the bottle contents?

One possibility may have to do with the use of recycled PET. In 2008, 27.0% of PET containers sold in the United States were recycled (National Association for PET Container Resources 2009). “New” PET may therefore contain PET that has been recycled from a previous use. PET recycling begins by washing the used PET to remove contaminants; however, this washing is not effective in removing organic molecules once they have been sorbed into the bottle wall (Safa 1999).

PET from different suppliers may differ in the degree to which it is homopolymer or copolymer, the extent to which the material is “virgin” or recycled PET, and in details of the manufacturing process. As noted above, Bošnir et al. (2007) detected DMP in concentrations as high as 3,000 μg/L in PET-bottled soda, whereas they were unable to detect DMP at all in PET-bottled mineral water. One possible explanation is that the soda, perhaps because of its lower pH, promoted leaching of DMP from the PET bottle wall. However, it is also possible that the PET used in production of the bottles intended for soda had a different provenance than the PET used in production of the bottles intended for mineral water. Shampoo often contains DMP (e.g., Sathyanarayana 2008). If the bottles used for soda included PET recycled from shampoo bottles, whereas the PET-bottled mineral water did not, that difference might contribute to the much higher concentrations of DMP in PET-bottled soda.

## Estrogenicity of Antimony, and Leaching of Antimony from PET

Measuring the concentration of phthalates in soft drinks and 3% acetic acid, respectively, Bošnir et al. (2007) and Farhoodi et al. (2008) both found phthalates in concentrations > 1,000 μg/L in at least some of their samples. However, among the other reports cited above that measured phthalates in bottled water rather than in soda or acetic acid, only one (Biscardi et al. 2003) identified any phthalates in concentrations > 1,000 μg/L, and that was only after at least 9 months of incubation. Nevertheless, the bioassays described at the beginning of this article incubated their specimens for < 2 months and employed water or a water-based culture medium with a neutral or near-neutral pH, not with soft drinks or acetic acid. This raises the possibility that a nonphthalate endocrine disruptor or disruptors may have mediated the estrogenic effects documented in the bioassays. Some evidence suggests that antimony may be at least partially responsible for these estrogenic effects.

Choe et al. (2003) reported that antimony chloride has “high estrogenicity” in two bioassays. In an estrogen-receptor–dependent transcriptional expression assay using human breast cancer cells, they found that 1 μM antimony chloride had estrogenic activity that was 61% equivalent to 1 nM 17β-estradiol. In an E-screen assay measuring proliferation of human breast cancer cells, they reported that 1 μM antimony chloride had estrogenic activity that was 49% equivalent to 10 nM 17β-estradiol.

The U.S. Environmental Protection Agency (EPA) has established an MCL (maximum contaminant level) of 6 ppb for antimony, which is the same limit set by Health Canada; the German Federal Ministry of Environment has set a limit of 5 ppb, whereas the Japanese drinking water standard requires levels of antimony < 2 ppb (Shotyk and Krachler 2007). However, these cutoffs are generally based on older research on antimony toxicity, related to cardiovascular risks and carcinogenicity; for example, the U.S. EPA’s Web site on antimony in drinking water makes no mention of antimony’s possible endocrine-disrupting effect (U.S. EPA 2009).

Antimony is widely used as a catalyst in the polycondensation of PET (Pang et al. 2006). PET resin typically contains antimony in concentrations between 100 and 300 mg/kg (Duh 2002). However, PET resin made in Japan is sometimes manufactured using titanium rather than antimony as a catalyst. Nishioka et al. (2002) investigated antimony concentrations in PET bottles manufactured in Japan. They found a roughly bimodal distribution of concentrations, with bottles from four manufacturers having mean antimony concentrations between 168 and 216 mg/kg, whereas in bottles from two other manufacturers, antimony concentrations were below the limit of detection (< 0.1 mg/kg); bottles from a seventh manufacturer had a mean antimony concentration of 58 mg/kg.

Several investigators have now demonstrated significant levels of antimony in water bottled in PET containers. Shotyk and Krachler (2007) measured antimony concentrations in 132 brands of bottled water purchased in 28 countries. They found a wide variation in antimony concentrations, with dramatic differences in the leaching of antimony over time. In 14 brands of PET-bottled water purchased in Canada, antimony concentrations increased on average 19% during 6 months of storage at room temperature. By contrast, 48 brands of PET-bottled water purchased in Europe increased on average 90% during 6 months of storage, under identical storage conditions in the same laboratory. Shotyk and Krachler also reported wide variations in antimony concentrations even among the same brand of PET-bottled water, depending on the location of purchase. For example, one brand of PET-bottled water yielded 1,650 ng/L of antimony when first purchased in Hong Kong, increasing to a concentration of 1,990 ng/L when tested 6 months later, whereas the same brand of bottled water purchased in Europe had a concentration of 725 ng/L when first purchased, increasing to 1,510 ng/L 6 months later.

Westerhoff et al. (2008) found that raising the ambient temperature significantly increases the leaching of antimony into nine brands of PET-bottled water purchased in the United States. At room temperature (22°C) they found no significant change in the concentration of antimony over time: The average antimony concentration from nine brands of PET-bottled waters was 0.195 ± 0.116 ppb at the beginning of the study and 0.226 ± 160 ppb after 3 months indoors at 22°C. When the bottles were incubated at 70°C, however, the concentration reached 6 ppb in just 12 days; at 80°C, in just 2.3 days. After 7 days at 80°C, the antimony concentration reached 14.4 ppb. Noting that temperatures within a closed-container truck may easily exceed 60°C in Arizona, where this study was conducted, Westerhoff et al. (2008) concluded that “short duration exposure to elevated temperatures during transit or storage by the seller or consumer could yield antimony concentrations that approach or exceed the 6 ppb MCL.” Previous research has demonstrated that the temperature inside a car parked in the sun, with windows closed, can reach 78°C after 6 hr (Surpure 1982).

Keresztes et al. (2009) studied 10 brands of PET-bottled water, all purchased in Hungarian supermarkets. Unlike Westerhoff et al. (2008), Keresztes et al. found that the concentration of antimony in PET-bottled water increased monotonically over time at room temperature, although they reported large differences between brands. The antimony concentration in one brand of PET-bottled water increased from an average initial concentration of about 0.1 ng/mL to an average concentration of about 0.9 ng/mL after 2 years’ incubation at room temperature. However, another brand of PET-bottled water showed almost no additional leaching of antimony even when warmed to 60°C for 24 hr (the concentration always < 0.2 ng/mL), whereas the concentration of antimony in a different brand of PET-bottled water increased from 0.2 ng/mL at 22°C to > 1.5 ng/mL after 24 hr incubation at 60°C (Keresztes et al. 2009, their figure 5).

## Discussion

The available research suggests that the concentration of phthalates in the contents of PET bottles varies as a function of the contents of the bottle, with phthalates leaching into lower pH products such as soda and vinegar more readily than into bottled water. Temperature also appears to influence the leaching both of phthalates and of antimony from PET, with greater leaching at higher temperatures.

The effect of temperature may account for some of the variation in the results noted previously. For example, Pinto and Reali (2009) noted that “cell toxicity was observed for water samples of the same lot of three different brands purchased from the same retailer”; they conjectured that “toxicity might be attributable to the storage conditions of the product.” Perhaps that retailer left the bottles exposed to the hot sun, whereas other retailers did not.

Lower-pH condiments such as table vinegar and salad dressing may warrant particular attention. The findings of Farhoodi et al. (2008) suggest that ingesting several servings of salad dressing that had been stored in a warm warehouse for a month might result in a dose of DEHP on the order of several hundred micrograms, possibly reaching the reference dose limit of 20 μg/kg/day (U.S. EPA 2006).

## Conclusion

The evidence suggests that PET bottles may yield endocrine disruptors under conditions of common use, particularly with prolonged storage and elevated temperature. Important questions for future research include the following: What substances in the water are responsible for the estrogenic effects observed in the bioassays—is it one or more of the phthalates, and/or antimony, and/or as yet unidentified substances? How do variations in the composition and manufacture of PET influence the leaching of these substances into the contents of the bottle? Would special measures—such as a special coating on the inner wall of the bottle (e.g., Pennarun et al. 2004), or transportation under controlled-temperature conditions—minimize the leaching of these substances into the contents? Because of the widespread use of PET plastic worldwide in containers for water, soda beverages, and condiments, the safety of PET under conditions of common use certainly merits further investigation.
